# Evaluation of the modified immune prognostic index to prognosticate outcomes in metastatic uveal melanoma patients treated with immune checkpoint inhibitors

**DOI:** 10.1002/cam4.3784

**Published:** 2021-03-16

**Authors:** Michael S. Sander, Igor Stukalin, Isabelle A. Vallerand, Siddhartha Goutam, Benjamin W. Ewanchuk, Daniel E. Meyers, Aliyah Pabani, Don G. Morris, Daniel Y. C. Heng, Tina Cheng

**Affiliations:** ^1^ Cumming School of Medicine University of Calgary Calgary AB Canada; ^2^ Tom Baker Cancer Centre University of Calgary Calgary AB Canada; ^3^ Section of Dermatology University of Calgary Calgary AB Canada; ^4^ Faculty of Medicine and Dentistry University of Alberta Calgary AB Canada

**Keywords:** biomarkers, melanoma, prognosis, prognostic factor, survival

## Abstract

**Background:**

Metastatic uveal melanoma (MUM) is associated with poor survival and inferior response to immune checkpoint inhibitor (ICI) therapy when compared with metastatic cutaneous melanoma. Currently, prognostic biomarkers are lacking to guide treatment decisions.

**Patients and Methods:**

We conducted a multicenter, retrospective cohort study using a centralized, province‐wide cancer database in Alberta, Canada. We identified 37 patients with histologically confirmed MUM who received at least one dose of single‐agent pembrolizumab or nivolumab, or combination therapy nivolumab and ipilimumab. A modified immune prognostic index (IPI), based on the previously reported lung immune prognostic index, was used to stratify patients into favorable and poor IPI groups. Survival analyses were conducted using the Kaplan–Meier method and Cox proportional hazards models, adjusting for baseline age (≥60) and ECOG performance status, to assess the associations between IPI and overall survival (OS). Time to treatment failure (TTF) was also assessed using the Kaplan–Meier method. The association between IPI and objective response rate was examined using chi‐squared tests. Logistic regression was used to determine the association between IPI and immune‐related adverse events (irAEs).

**Results:**

Median OS was 15.6 (range 0.6–57.6) months with 45.9% 1‐year survival rate at a median follow‐up of 11.8 months. We found that a favorable IPI was significantly associated with OS [median 30.5 (12.0‐not reached) months in the favorable IPI group compared with 4.6 (2.1–16.0) months in the poor IPI group (*p* = 0.001)] (HR=4.81, 95% CI; 1.64–14.10, *p* = 0.004), TTF [median 5.1 (95% CI; 2.1–10.4) months in the favorable IPI group compared with 3.7 (95% CI; 1.4–6.4) months in the poor IPI group (*p* = 0.0191)], and irAE (HR=6.67, 95% CI; 1.32–33.69, *p* = 0.0220).

**Conclusions:**

The modified IPI may be a useful tool in clinical practice for identifying MUM patients who are more likely to experience irAEs and realize a survival benefit from ICI treatment.

## Graphical abstract

The modified immune prognostic index (IPI) for metastatic uveal melanoma dichotomizes patients into favorable and poor IPI cohorts that have significantly different median overall survival when treated with immune checkpoint inhibitors. The IPI is calculated using commonly ordered laboratory tests, and can be a valuable prognostic tool to assist with individualized treatment planning

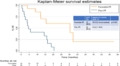

## 1 INTRODUCTION

Uveal melanoma (UM) accounts for about 3% of all melanoma. It is a subtype of sun‐shielded melanoma arising from melanocytes located in the choroid, ciliary body, and iris of the eye. UM is distinct in molecular pathogenesis, with ultraviolet radiation‐induced signature mutations in the cancer genomes restricted to iris UM,^1^ and a median count of nine somatic mutations per tumor compared with a median of 171 somatic mutations in cutaneous melanoma (CM).^2^ Driver mutations are also distinct, commonly affecting GNAQ or GNA11, while mutations in BRAF and NRAS, commonly seen in CM, are typically absent.^3^ UM is an aggressive malignancy that is associated with a poor prognosis; approximately half of all patients develop metastases, which spread hematogenously and predominantly to the liver.^4^, ^5^ When metastases occur, UM is typically fatal within 1 year.^6^

Prior to 2010, the prognosis for all subtypes of metastatic melanoma (MM) was poor, with no effective systemic therapies.^7^ The median overall survival (OS) of patients with metastatic cutaneous, uveal, acral, and melanoma of unknown primary were all similar and ranged between 10 and 13 months.^8^ In the last decade, significant advances have been made in the treatment of metastatic cutaneous melanoma (MCM) in the form of immune checkpoint inhibitors (ICI). ICI used in the treatment of MCM include antibodies directed against programed cell death protein 1 (PD‐1) such as nivolumab and pembrolizumab, anticytotoxic T‐lymphocyte antigen‐4 (CTLA‐4) agents like ipilimumab, or combination regimens of such. Remarkably, objective response rates (ORR) as high as 61.0%^9^ and median OS beyond 60.0 months ^10^ have been demonstrated in MCM clinical trials with combined nivolumab and ipilimumab.

Although MUM patients have typically been excluded from large MCM clinical trials, systemic treatment regimens for MUM are often still guided by treatment recommendations for MCM.^11^ Prospective phase II clinical trials and retrospective studies looking at MUM outcomes reported ORR between 2.6% and 21.0%, and median OS between 5.0 months and not reached, with the longest median OS being 18.9 months with ORR at 21% using combination ipilimumab and nivolumab.^12^ The extent to which ICI impact survival is still unclear. Data on the efficacy of ICI in treating MUM are mostly retrospective in nature, with a few small prospective phase I and II studies. However, more recent studies suggest anti‐PD1 agents are superior over anti‐CTLA‐4 agents in treating MUM, and combination regimens that expose patients to both classes of antibodies may lead to higher response rates, and possibly long‐term survival.^6^, ^11^, ^13^, ^14^

When comparing outcomes of MUM and MCM patients treated with ICI, ORR and OS are both significantly lower in MUM, and the ability to predict which MUM patients will respond favorably to treatment is limited. Prognostic markers are needed to identify those MUM patients who are likely to benefit from treatment.^15^, ^16^ The Lung Immune Prognostic Index (LIPI) was originally described by Mezquita et al.^17^ as a prognostic tool for patients with non‐small cell lung cancer (NSCLC). The LIPI is based on measurements of serum lactate dehydrogenase (LDH) and a derived neutrophil to lymphocyte ratio (dNLR). An LDH greater than the upper limit of normal, and a dNLR greater than 3 were both independently associated with reduced OS (HR, 2.44; 95% CI, 1.47–4.04, *p* = 0.001, and HR, 1.98; 95% CI 1.27–3.10, *p* = 0.002, log‐rank test). In creating the LIPI, Mezquita et al. stratified patients into poor (3.0 months; 95% CI 1.0 – not reached), intermediate (10.0 months, 95% CI 8.0 – not reached), and good (34.0 months; 95% CI 17.0 – not reached) (*p* < 0.001) LIPI groups, which were shown to be strongly associated with OS. The LIPI has been subsequently validated for patients with NSCLC, renal cell carcinoma (RCC), and MCM.^18^, ^19^, ^20^ The LIPI has the advantage of being easy to calculate since it relies on laboratory investigations that are routinely ordered for patients prior to starting ICI. Given the prognostic value of the LIPI in NSCLC, RCC, and MCM, we sought to determine whether a modified version of this index could be used in the prognostication of MUM. The modified immune prognostic index (IPI) is based on the previously described LIPI. Like the LIPI, the modified IPI uses LDH and dNLR to stratify patients into prognostic groups. The IPI differs from the LIPI in that two groups, favorable IPI and poor IPI, are discerned, whereas the LIPI score uses three groups: good, intermediate, and poor LIPI. The primary goal of our study was to assess the outcomes of MUM patients treated with ICI and determine if the modified IPI was prognostic of OS. Secondary goals included assessing whether the IPI was prognostic of other important outcomes, such as time to treatment failure (TTF) or immune‐related adverse events (irAEs).

## 2 METHODS

### 2.1 Study design

We conducted a retrospective cohort study at two tertiary cancer centers in Canada—the Tom Baker Cancer Centre in Calgary, Alberta and the Cross Cancer Institute in Edmonton, Alberta—using a centralized, province‐wide cancer database. We identified 496 MM patients who had received at least one dose of single‐agent pembrolizumab or nivolumab, or combination therapy with nivolumab and ipilimumab. Inclusion criteria for the study were as follows: age >18 years at time of metastatic disease diagnosis, histologically confirmed UM, and initiation of ICI therapy (nivolumab, pembrolizumab, or ipilimumab/nivolumab) between 1 January 2010 and 31 December 2019. Thirty‐seven patients satisfied the inclusion criteria. Patients were identified using consecutive provincial pharmacy records. The data collection and chart review occurred between 1 July 2017 and 30 April 2020. Individual retrospective chart reviews, using standardized database templates, were used to collect patient data. Pretreatment data were collected at the start date of ICI therapy. If a particular data point was not available in the range of 30 days prior to initiating ICI, the data point was deemed unavailable. Approval for the study was obtained through the Health Research Ethics Board of Alberta – Cancer Committee (HREBA. CC‐17–0215). Individual patient consent was not required due to the retrospective nature of this study.

Given our small sample size, we constructed a dichotomized version of previous ordinal LIPI scores.^17^, ^20^ The IPI score was calculated as poor IPI if a patient had either of the following two factors: (i) an LDH great than the upper limit of normal (>235 U/L) or (ii) a dNLR greater than 3.0. The dNLR was calculated as:

$$\frac{\left(\text{absolute neutrophil count}\right)}{\left(\text{total leukocyte count}‐\text{absolute neutrophil count}\right)}$$

A favorable IPI score was defined as having both an LDH below the upper limit of normal, and a dNLR less than 3.0.

Each patient's best overall radiological response was assessed by the site investigator based on the Response Evaluation Criteria in Solid Tumors (RECIST) v1.1. As part of routine clinical care, patients were staged with CT and/or MRI scans prior to the initiation of ICI and approximately every 12 weeks thereafter. Patients who did not receive a minimum of three ICI cycles, and/or who did not undergo diagnostic imaging restaging 12 weeks after treatment initiation did not have their best response assessed. Toxicity data were obtained from the patient's medical records and were graded using the National Cancer Institute Common Terminology Criteria for Adverse Events (CTCAE) v5.0. IrAEs are toxicities that are immune mediated in mechanism.

### 2.2 Statistical analysis

The primary endpoint of our study was OS, defined as the time from initiation of immunotherapy until the time of death from any cause, or the last known patient follow‐up. Observations were right censored when patients did not die during the study period. Person‐time was accumulated by patients until their last known follow‐up date. Secondary outcomes included TTF, ORR, disease control rate (DCR), and the development of irAE events. ORR was defined as the proportion of patients who achieved a complete response (CR) or partial response (PR). The DCR was defined as the proportion of patients with a CR, PR, or stable disease (SD). TTF was calculated from the date of immunotherapy initiation until treatment discontinuation, due to radiologic progression, clinical deterioration, or death from any cause. Adverse events of all grades were recorded.

Survival analyses were conducted using the Kaplan–Meier method with log‐rank tests to compare survival curves across IPI groups. We also constructed Cox proportional hazards models to examine covariate effects on the association between IPI and OS. Hazard Ratios and 95% confidence intervals with p‐values from Wald tests were reported for each variable's association with OS. Respecting 10 events per variable rule of thumb for multivariate modeling in prognostic models of cancer,^21^ we constructed models of IPI on OS accounting for baseline age (<60 or ≥60),^22^ and Eastern Cooperative Oncology Group (ECOG) performance status.^20^ Interaction terms were created between IPI and age, and also IPI and ECOG. The presence of statistical interactions (effect modification) was assessed using Wald tests. A backward elimination process was then used whereby a full model was first constructed, and each of the covariates were then removed from the model, one at a time, to observe the resulting effect on the estimated hazard ratio. A >10% change to the estimated HR was deemed to be suggestive of a confounding effect. A final model was constructed with the goal of adjusting for any confounding effects, and a parsimonious model was reported without including redundant variables that do not contribute to OS. The proportional hazards assumption was assessed using Schoenfeld residuals and log–log plots.

The association between IPI and ORR and DCR was assessed using chi‐squared tests. Logistic regression with odds ratios (OR) and 95% confidence intervals was used to examine the relationship between IPI and the development of irAEs. The Kaplan–Meier method with Wilcoxon rank‐sum tests was used to compare median OS time, and was also used to compare median TTF across IPI groups.

Patients missing data (*n* = 6) were excluded from multivariate analyses. However, we conducted a sensitivity analysis whereby missing values of either LDH or dNLR (required to calculate an IPI score) were imputed with multiple imputation using 20 iterations (*m* = 20), and Cox proportional hazards regression was used to determine the association between an imputed IPI and OS. Furthermore, we also conducted a sensitivity analysis to examine the relationship between the previously reported ordinal IPI score and OS, where IPI is defined as a total of two factors with a score of 0, 1, or 2, corresponding to favorable, intermediate, and poor IPI, respectively.^17^ A *p*‐value of <0.05 defined statistical significance for all analyses. The statistical analyses were performed using Stata v14.2 (College Station, Texas, USA).

## 3 RESULTS

### 3.1 Patient demographics

Demographics and clinical courses of the 37 MUM patients who received ICI are summarized in Table 1. The median age at diagnosis of UM was 59.2 years (IQR: 9.5 years). Twenty‐seven patients (73.0%) had favorable ECOG scores (0 or 1). Overall, 15 (40.5%) MUM patients were classified as having a favorable IPI, 16 (43.2%) were classified as having a poor IPI, and five (16.2%) had missing data and were not able to be classified.

**TABLE 1:** Baseline demographics of the patient population

	*N* = 37 (%)
Gender	
Male	21 (56.8)
Female	16 (43.2)
Age at initial diagnosis	
<60 years	20 (54.1)
≥60 years	17 (45.9)
ECOG status	
0	14 (37.9)
1	13 (35.1)
2	7 (18.9)
3	3 (8.1)
Metastatic sites	
Liver	32 (86.5)
Lung	14 (37.9)
Bone	3 (8.1)
Brain	2 (5.4)
Other	3 (8.1)
Serum LDH	
Normal	16 (43.2)
Elevated	15 (40.6)
Unknown	6 (16.2)
dNLR	
≤3.0	32 (86.5)
>3.0	5 (13.5)
IPI Score	
Favorable	15 (40.6)
Poor	16 (43.2)
Unknown	6 (16.2)

Twenty‐one (56.8%) patients received pembrolizumab (2 mg/kg intravenously every 3 weeks), seven (18.9%) received nivolumab (3 mg/kg intravenously every 2 weeks), and nine (24.3%) received combination ipilimumab and nivolumab (ipilimumab 3 mg/kg and nivolumab 1 mg/kg intravenously every 4 weeks for up to four cycles, followed by nivolumab 3 mg/kg intravenously every 2 weeks). The median number of ICI cycles received was 6.5, and the median follow‐up from the date of ICI initiation to the last clinical encounter was 11.8 months. At the end of the study period (April 30, 2020), 13 (35.1%) patients were still alive.

### 3.2 Overall survival

**FIGURE 1:**
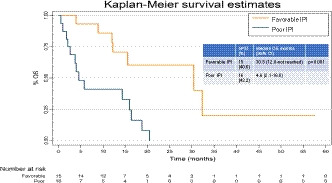
Kaplan‐Meier curve depicting overall survival of patients with favorable and poor immune prognostic index (IPI) scores.

The median OS was 15.6 (95% CI; 6.9–20.5) months, with a range of 0.6 to 57.6 months. The survival rate was 45.9% at 1 year and 13.5% at 2 years. When stratified by IPI status, the OS was 30.5 (12.0‐not reached) months and 4.6 (2.1–16.0) months in the favorable IPI and poor IPI groups, respectively (*p* = 0.001) (Figure 1). There were no statistically significant differences in median OS between patients on single‐drug ICI (14.4 months, 95% CI; 6.1–20.5) compared with combination therapy (16.0 months, 95% CI; 3.8‐not reached) (*p* = 0.40).

In multivariate analyses, a significant association was observed between IPI and OS (HR = 4.88, 95% CI; 1.73–13.78, *p* = 0.003). No significant interactions were observed between IPI and age (*p* = 0.135) or IPI and ECOG status (*p* = 0.180). The association between IPI and OS remained significant after adjusting for potential confounding effects of age and ECOG status (HR = 4.81, 95% CI; 1.64–14.10, *p* = 0.004) (Table 2). There was no evidence of a violation of the proportional hazards assumption (*p* = 0.911).

**TABLE 2:** Multivariate Cox regression analysis

	Category	HR	*p*‐value	95% CI
Crude model				
IPI	Favorable	1		
	Poor	4.88	0.003*	1.73–13.78
Adjusted model				
IPI	Favorable	1		
	Poor	4.81	0.004*	1.64–14.1
Age	<60	1		
	≥60	2.12	0.130	0.80–5.62
ECOG Status	<2	1		
	≥2	1.62	0.336	0.60–4.37

Three parameters were included in the multivariate Cox regression analysis. Of these factors, only the IPI classification was significantly associated with overall survival in this model.

Abbreviations: CI, confidence interval; ECOG, Eastern Cooperative Oncology Group; HR, hazard ratio, IPI, Immune Prognostic Index.

* *p* <.05

### 3.3 Objective response rate and disease control rate

The ORR was 5.4%, notable for two patients who achieved a PR. No patients achieved a CR. The DCR was 32.4%, with 12 patients having attained either a PR or SD (Table 3). There was no significant association between IPI status and ORR (*p* = 0.189) or for IPI status and DCR (*p* = 0.137).

**TABLE 3:** Summary of best responses to treatment with single or combination ICI

	***N* = 37 (%)**
Complete response	0 (0.0)
Partial response	2 (5.4)
Stable disease	10 (27.0)
Progressive disease	21 (56.8)
Not available	4 (10.8)
Overall response rate	2 (5.4)
Disease control rate	12 (32.4)
	**Months (95% CI)**
Overall survival, median	15.6 (6.9–20.5)
Time to treatment failure, median	4.3 (3.1–5.3)

### 3.4 Time to treatment failure

Patients with a favorable IPI status had a statistically significant longer time until treatment failure, with a median of 5.1 (95% CI; 2.1–10.4) months, compared to patients with a poor IPI status, who remained on treatment for a median of 3.7 (95% CI; 1.4–6.4) months (*p* = 0.0191). Only five (13.5%) patients remained on treatment for at least 12 months, and two (5.4%) received 24 months or more of ICI therapy.

### 3.5 Adverse events

In total, 19 (51.4%) patients reported an irAE. Details relating to the specific type and severity of irAEs are outlined in Table 4. Combination ICI therapy with nivolumab plus ipilimumab was statistically significantly associated with the development of irAE (*p* = 0.01). Eight of nine (88.9%) patients on combination treatment developed an irAE, compared to only 11 of 28 (39.3%) patients on single‐agent ICI. Patients who experienced an irAE had a statistically significant longer survival compared to patients who did not experience an irAE (median 16.0 months, 95% CI; 12.0–32.3 vs median 6.9 months, 95% CI; 2.6–23.2, *p* = 0.0138). Furthermore, patients with a favorable IPI status were more likely to have an irAE than those with poor IPI status (*p* = 0.017). Logistic regression demonstrated a statistically significant association (HR = 6.67, 95% CI; 1.32–33.69, *p* = 0.0220) between IPI and the development of an adverse event.

**TABLE 4:** Adverse events observed during treatment with single or combination ICI

Toxicity	Grade 1 (%)	Grade 2 (%)	Grade 3 (%)	Total (%)
Diarrhea/colitis	2 (2.7)	4 (10.8)	2 (5.4)	8 (21.6)
Transaminitis	3 (8.1)	2 (5.4)	3 (8.1)	8 (21.6)
Rash	6 (16.2)	1 (2.7)	0 (0.0)	7 (18.9)
Pruritus	6 (16.2)	0 (0.0)	0 (0.0)	6 (16.2)
Hypothyroid	0 (0.0)	5 (13.5)	0 (0.0)	5 (13.5)
Thyroiditis	1 (2.7)	1 (2.7)	1 (2.7)	3 (8.1)
Vitiligo	3 (8.1)	0 (0.0)	0 (0.0)	3 (8.1)
Hypophysitis	0 (0.0)	0 (0.0)	0 (0.0)	2 (5.4)
Pneumonitis	0 (0.0)	1 (5.4)	1 (5.4)	2 (5.4)
Myositis	1 (2.7)	1 (2.7)	0 (0.0)	2 (5.4)

Four patients experienced adverse events in addition to those listed above. These included one case each of diabetes, elevated amylase, hyperbilirubinemia, and uveitis.

### 3.6 Sensitivity analyses

The association between IPI and OS remained significant even after imputing missing values (*n* = 6) for LDH or DNLR to calculate IPI (HR = 4.55, 95% CI; 1.76–11.78, *p* = 0.002). When IPI was assessed as an ordinal variable with three levels, the median OS time for patients with a favorable IPI status was 30.5 (95% CI; 8.9–not reached) months, with an intermediate IPI was 14.4 (95% CI; 3.8–18.8) months, and with a poor IPI status was 1.0 (95% CI; 0.6‐not reached) months. The association between the ordinal IPI and OS also remained significant (HR = 5.14, 95% CI; 2.23–11.88, *p* < 0.0001).

## 4 DISCUSSION

Our study identified a highly prognostic index to predict OS in MUM patients receiving ICI. Previous studies attempting to develop prognostic indices for MUM patients receiving ICI were more complex, used a greater number of clinical parameters, and had a reduced ability to discriminate survival.^22^ The modified IPI score uses LDH and dNLR to stratify patients into two prognostic groups—favorable IPI and poor IPI. As the median survival for patients in the favorable IPI cohort was 30.4 months versus 4.6 months in the poor IPI group, the IPI score was able to discriminate OS with a greater than 2‐year difference between groups. The IPI prognostic tool is readily available to the physician to prognosticate and counsel MUM patients, and also to stratify patients participating in clinical trials.

It is hypothesized that MUM is less responsive to ICI due to the presence of fewer somatic mutations, which results in fewer potential neoantigens that can be targeted by antitumor immunity. Additionally, the liver as an immune‐modulatory organ may protect UM metastases from immune surveillance.^6^ However, UM expresses several immunogenic antigens, such as glycoprotein 100 (gp100), melanoma antigen recognized by T cells (MART‐1), and tyrosinase,^23^ and a subset of UM are able to elicit a vigorous immune response.^24^ Rare marked response to immunotherapy has been reported in UM, and molecular investigation of these tumors revealed high tumor mutation burden (TMB) secondary to germline, loss‐of function MBD4 mutations.^13^, ^14^ Johansson et al. recently demonstrated that iris UM is unique among UM subtypes in that it demonstrates ultraviolet radiation‐associated DNA damage, and like MBD4‐deficient tumors, has a high TMB.^1^ This suggests that iris UM may be more likely to respond to immunotherapy than other variants of UM, the vast majority of which have low TMB.

Wessely et al. ^12^ recently reviewed the evidence for ICI in treating MUM. Among nine prospective and retrospective studies using anti‐CTLA‐4 (ipilimumab or tremelimumab) for the treatment of MUM, the largest prospective observational study with 53 patients reported ORR of 0% and a median OS of 6.8 months. The largest retrospective study with 82 patients reported ORR of 4.8% and a median OS of 6.0 months. Among 11 retrospective and prospective studies utilizing anti‐PD1 agents (pembrolizumab or nivolumab), the largest prospective study had 34 evaluable patients and reported ORR of 5.8% and median OS of 11 months for patients on nivolumab. The largest retrospective study which followed 43 patients on pembrolizumab reported ORR of 7.0% and a median OS of 10.3 months. Improved responses are reported with combined ICI. One retrospective study consisting of 64 patients reported ORR of 15.6% (3.1% CR) and a median OS of 16.1 months,^22^ while another with 89 patients reported ORR of 11.0% (1% CR) and a median OS of 15.0 months.^25^ Bol et al. reported the longest median OS in the literature at 18.9 months for combination ipilimumab and nivolumab, with an ORR of 21.0%, albeit with a small sample size of 19 patients.^26^ A recent study by Klemen et al. followed 30 MUM patients treated with ICI. The study had four patients survive >5 years, all of whom received anti‐CTLA‐4 and anti‐PD1, either sequentially or in combination. The author suggested that exposure to ipilimumab in addition to anti‐PD1 may be integral in achieving long‐term survival in MUM.^7^ Collectively, data suggest that combined ICI may be superior to anti‐PD1 or anti‐CTLA‐4 monotherapy, although we do caution that there are limitations in the current data with small sample size, potential selection bias, and a lack of clinical trials with comparative study design. In our study, we did not find any significant differences in OS or ORR between MUM patients on single versus combination therapy, likely due to small sample size.

To date, a number of prognostic markers have been explored in MCM with varying success. In patients with CM, C‐reactive protein (CRP) has been shown in several studies to be an independent prognostic marker.^27^, ^28^, ^29^ CRP has a high degree of sensitivity for detecting progression from stage III to IV MCM, as well as for predicting survival outcomes in patients with stage IV MCM.^27^, ^28^, ^29^ In the setting of MUM, however, the utility of CRP as a prognostic factor is less convincing.^22^, ^30^ Additional prognostic markers that have shown potential value in patients with MCM are the pretreatment expression of programmed cell death ligand 1 (PD‐L1) and the tumor mutation burden. In patients with MCM, several studies have demonstrated a positive association between PD‐L1 expression in the tumor microenvironment and OS, progression‐free survival (PFS), and ORR.^31^, ^32^ TMB has been found to be significantly higher in ICI responders, and also associated with a survival benefit.^33^ Unfortunately, neither PD‐L1 expression or TMB appears to be of prognostic value in patients with MUM.^34^, ^35^ Finally, several retrospective analyses have demonstrated that tumor thickness and chromosome 3 alterations may be used as markers to identify MUM patients at high risk of rapid disease progression.^36^, ^37^ However, these tests have not been implemented into routine clinical practice for either risk stratification or treatment planning.^36^, ^37^ The advantages that the IPI score carries over other prognostic markers include being simple to calculate, using blood markers that are already routinely obtained from patients prior to ICI treatment, and demonstrating a marked ability to dichotomize patients into groups with significantly different median OS.

The IPI is a significant prognostic index to predict OS in MUM patients receiving ICI; however, it is not a predictive index for treatment response to immunotherapy. A prognostic marker provides information about the patient's overall outcome, regardless of therapy. A predictive marker gives information about the effect of a particular therapeutic intervention, such as high TMB in melanoma predicting response to immunotherapy. Although there is a significant difference in OS between IPI cohorts, we did not find an association between IPI score and ORR or DCR, and therefore cannot say based on the available data that the IPI is predictive of treatment response. The most likely explanation for the lack of association between IPI score and ORR or DCR is the small sample size of our study combined with the low ORR and DCR seen in MUM. With only two patients experiencing a PR to treatment, and only 10 others having SD, the ability to statistically demonstrate an association between IPI score and treatment response is limited. Interestingly, patients with a favorable IPI status had a significantly longer TTF (5.1 months, 95% CI; 2.1–10.4) compared to patients with poor IPI status (3.7 months, 95% CI; 1.4 – 6.4). This suggests that the quality of disease control may be superior in the favorable IPI group, leading to better survival compared to the poor IPI group.

In our complete patient cohort, just over half (51.4%) of patients experienced an irAE, which is similar to previous studies of MUM patients, which have reported irAE rates between 25.0% and 60.0%.^22^, ^25^, ^30^ We also found that the rate of irAE in the favorable IPI group was significantly higher than in the poor IPI group: 63.0% versus 20.0%, respectively. Since normal tissue antigens and tumor neoantigens may be cross‐reactive, immune‐related irAE may in fact indicate a robust immunological response to ICI, potentially correlating with antitumor response.^38^ Previous studies have demonstrated a positive association between irAE and antitumor efficacy.^39^, ^40^ Additionally, Suo et al.^41^ reported anti‐PD1‐related irAE to be associated with significantly improved OS. We found a strong association between favorable IPI (improved median OS) and irAE development (HR = 6.67, 95% CI; 1.32–33.69, *p* = 0.0220), further lending support to there being a modest treatment effect of ICI.

A limitation of our study was the small sample size of 37 patients. However, despite the small sample size, our study maintained sufficient statistical power to detect a significant association between IPI and our primary outcome of interest, OS. Our study was also limited by being a nonrandomized, retrospective, observational study. Thus, the possibility of residual confounding exists, particularly as we were limited by sample size in our ability to adjust for potential confounders. However, the IPI is a highly objective measure, which limits the introduction of measurement bias. Although we were missing data to calculate IPI status in six (16.2%) patients, the results of our sensitivity analysis demonstrated a persistently significant association between IPI and OS when missing IPI values were imputed.

To our knowledge, our study is the first to investigate a modified version of the LIPI score, the modified IPI, for prognostication of patients with MUM. Future studies should be undertaken using larger MUM cohorts to further evaluate the utility of the IPI score for prognostication and to further validate the score. It would also be worthwhile to investigate whether the IPI score can be used for prognostication of other malignancies. A potential area of future research could be assessing whether favorable IPI patients can be further stratified using novel molecular prognostic factors in order to help determine individualized, best‐care pathways for each patient.

## CONFLICT OF INTEREST

The authors have declared no conflict of interest.

## AUTHORS’ CONTRIBUTIONS

Conceptualization: MSS, IS, DEM, DYCH, and TC. Data curation: MSS, IS, SG, BWE, and DEM. Formal analysis: MSS, IS, and IAV. Funding acquisition: TC. Resources and supervision: AP, DYCH, and TC. Writing – original draft: MSS, IS, IAV, and TC. Writing – review and editing: all the authors.

## Data Availability

All data relevant to the study are included in the article. De‐identified datasets used and/or analyzed during the current study are available from the corresponding author on reasonable request.
